# Longevity in the Different States

**Published:** 1885-05

**Authors:** 


### Longevity in the Different States.

A student of the reports of the tenth census has compiled a table for the Boston *Commonwealth* for the purpose of showing in what State or States one has the best chance for a long life. New Hampshire seems to him to be the favorite refuge of green old age, for he finds that one seventy-fourth of the inhabitants are at least eighty years old. The proportion among native white males is 1 to 80, but the environment in New Hampshire seems to have been even more favorable to the preservation of life in the other sex, for the proportion among native white males is 1 to 58. Other New England States do not contain quite so many old persons, the average proportion for the six being 1 in 134. Coming to New York, he finds that for one person who has reached the age of eighty there are 161 who have not been so fortunate, and in the three Middle States the average proportion is one in 182. As he goes southward he discovers a greater preponderance of young blood, for in six South Atlantic States the average proportion is 1 in 203. The Gulf States afford a less attractive shelter for the aged, for the average is 1 in 300. In Texas, where so many worthy persons die with their boots on in the prime of life, only one octogenarian can be found in a group of 497 citizens. The average rises again in the interior States east of the Mississippi, but in the Great Lake States it falls to 1 in 263, a good old age being attained with the greatest difficulty in the wealthy and prosperous State of Illinois. In seven States west of the Mississippi River the aged rarely appear, for the average proportion is 1 in 453. In Iowa a crop of 334 person yields only one who has reached the age of four score. In Minnesota, Nebraska, and Kansas only one of these aged citizens can be found in a group that would yield two in Iowa, and in Colorado 1,150 inhabitants must pass in review before an octogenarian comes in sight. The old are even more rare in Nevada, but in California and Oregon the proportion is nearly 1 in 500. If the inhabitants of the whole country could be assembled in two hundred and twenty-seven groups, it would be possible to place at the head of each group one patriarch of eighty or more years. So our student, assuming that long life is the inalienable right of those who reside in New Hampshire, Vermont and Maine, cries: "Flee to the mountains of New England for health and longevity!"—*Scientific American.*

